# Supplementary material for: Two-Dimensional MFI Zeolite Nanosheets Exfoliated by Surfactant Assisted Solution Process
Source: Nanomaterials (Basel). 2021 Sep 7;11(9):2327. doi: 10.3390/nano11092327 (PMC8472291; doi:10.3390/nano11092327)
Supplement: Supplementary file 1 [file nanomaterials-11-02327-s001.zip › nanomaterials-1351706-supplementary.pdf]

nanomaterials

Supplementary Materials

# Two-Dimensional MFI Zeolite Nanosheets Exfoliated by Surfactant Assisted Solution Process

**Aafaq ur Rehman [1,†], Devipriyanka Arepalli [1,†], Syed Fakhar Alam [1], Min-Zy Kim [1], Jungkyu Choi [2] and Churl Hee Cho [1,*]**

[1] Reaction & Separation Nanomaterials Laboratory, Graduate School of Energy Science and Technology, Chungnam National University, 99 Daehak-ro, Yuseong-gu, Daejeon 34134, Korea; aafaqktk@cnu.ac.kr (A.u.R.); devipriya0508@o.cnu.ac.kr (D.A.); fakhar9689@o.cnu.ac.kr (S.F.A.); mzkim@cnu.ac.kr (M.-Z.K.)

[2] Department of Chemical and Biological Engineering, College of Engineering, Korea University, 145, Anam-ro, Seongbuk-gu, Seoul 02841, Korea; jungkyu_choi@korea.ac.kr

* Correspondence: choch@cnu.ac.kr;

† These authors contributed equally to this work.

## 1. Experimental

### *1.1. Chemical Materials*

1-bromodocosane ($C_{22}H_{45}Br$, >98 %, TCI Chemical), 1-bromohexane ($C_6H_{13}Br$, >98%, TCI Chemical), *N,N,N′,N′*-terametheyl-1,6-diaminohexane ($C_{10}H_{24}N_2$, >98% TCI Chemical), acetonitrile (ACS reagent, Sigma Aldrich), diethyl ether (ACS reagent, Sigma Aldrich), toluene (extra pure, Sigma Aldrich), tetraethyl orthosilicate (TEOS, 98%, Sigma Aldrich), sodium hydroxide (NaOH, 99%, Junsei Chemical), polyvinylpyrrolidone (PVP), *n*-butanol, chloroform, *ortho*-dicholorobenzene (ODCB) and *N′* dimethyl formamide (N′ DMF) were purchased from Sigma Aldrich and used as received.

### *1.1. Synthesis of di-quarternary ammonium structural direction agent*

In this study, the bi-functional organic surfactant (di-quaternary ammonium type, $C_{22\text{-}6\text{-}6}$-Br) was synthesized by the method developed by Ryoo and co-workers [1]. In typical synthesis of organic structure-directing agent (OSDA) for generating multilamellar silicalite-1 nanosheets (MLSil-1Ns), 0.2 mol of 1-bromodocosane and 2 mol of *N, N, N′, N′*-tetramethyl-1,6-diaminohexane were dissolved in 100 mL of toluene/acetonitrile mixture (1:1 *vol/vol*) and slowly heated in three mouth round bottom flask under refluxing attached with condenser at 70 °C for 12 h. The solid product was collected at the bottom of flask followed by filtration and washed with cold diethyl ether and dried at 50 °C under the vacuum. An amount of 0.2 mol of the product obtained in the first step was slowly added to the solution of 0.2 mol 1-bromohexane and 30 mL acetonitrile and subsequently refluxed at 80 °C for 12 h. The obtained product on cooling resulted in precipitated products that were filtered, washed with cold diethyl ether and dried at 50 °C overnight under the vacuum. Approximately 5 g of the product was dissolved in 30 g of DI $H_2O$ and stirred for 24 h to yield 10-15% aqueous OSDA to exchange the $C_{22\text{-}6\text{-}6}$-Br ($Br^-$) to $C_{22\text{-}6\text{-}6}$-OH ($OH^-$). After 24 h, the exchanged solution was filtered and bromine test was conducted to determine the complete removal of $Br^-$ ion in the aqueous solution. The obtained product after the exchange was used as final OSDA for the synthesis of MLSil-1Ns.

### *1.3. Synthesis and Exfoliation of multi-lamellar silicalite-1 nanosheets*

In a typical synthesis of MLSil-1Ns, 4.17 g of TEOS was added dropwise to the 13.4 g of aqueous solution of $C_{22\text{-}6\text{-}6}$-OH and stirred at room temperature for 0.5 h. The gel solution was cooled on ice bath for 0.5 h. After cooling on an ice bath for 0.5 h, an additional 5.0 g of DI $H_2O$ was added dropwise into the solution and stirred at room temperature for 6 h. The final molar composition of synthesis gel was $1SiO_2$: $0.13C_{22\text{-}6\text{-}6}$-OH: $60H_2O$. After aging

for 6 h, the solution was transferred to PTFE-lined stainless-steel autoclave and slowly heated to and maintained at 150 °C for different periods (5-15 d) under static condition. The product obtained after varying time was denoted as MLSil-1Ns-x, where "x" is the number of hydrothermal days. After the crystallization of MLSil-1Ns, the autoclave was removed from the oven and the solid product at the bottom of the autoclave was recovered by filtration, extensively washed with DI $H_2O$ by centrifugation in eight 50 mL centrifuge tubes for two times using centrifuge (Supra 22K, equipped with A50S-8 rotor) at 18,000 rpm for 20 min. Finally, it was dried at 60 °C overnight under vacuum. The dried product was calcined at 550 °C for 5 h to remove the OSDA located in the crystalline materials.

Exfoliation of MLSil-1Ns was performed by mild milling in the presence of PVP as a surfactant. PVP is highly stable in aqueous solution [2]. In typical exfoliation of MLSil-1Ns, firstly a 10 wt.% stable suspension of PVP was separately prepared in DI $H_2O$. In next step, zeolite nanosheets were added in the surfactant suspension in the ratio of 0.16 g of zeolite nanosheets and 3.84 g to 10 wt.% surfactant suspension and sonicated for 1 h using sonicator (Powersonic 605, 350 watts). The sonicated samples were subjected to rapid exfoliation under mild planetary milling of 400 rpm for 15 min (FRITSCH Ball mixer/mill15).

In typical purification process, the exfoliated solution with PVP was dispersed in toluene to 1~5 wt.% suspension by sonication for 1 h. The resulting solution was centrifuged in two 50 mL centrifuge tubes at 18,000 rpm for 40 min to sediment the MLSil-1Ns at the bottom of the centrifuge tubes. Sedimented MLSil-1Ns were slowly and carefully separated from the supernatant and re-dispersed in toluene. This process was repeated three times to completely remove the injected PVP in the nanosheets. Finally, the sedimented MLSil-1Ns were obtained by pouring out the supernatant and bottom products were dispersed in organic solvating agents such as *n*-butanol, chloroform and *ortho*-dichlorobenzene (ODCB) and *N'N'* dimethylformamide to observe the suspension quality. Furthermore, to check the dispersible exfoliated nanosheets in the suspension for easy fabrication of thin films, characterization and quantification of nanosheets were performed to check the effect of various parameters.

### *1.4. Characterization of Materials*

Powder X-ray diffraction (XRD) patterns were obtained using PAN analytical X-pert Pro diffractometer with Cu K$\alpha$ radiation. Nitrogen adsorption and desorption isotherm were measured using BELSORP-max Japan Inc., at the liquid $N_2$ temperature of 77.35 K. Before the adsorption analysis, the samples were outgassed at 300 °C for 12 h under vacuum. The BET surface area and pore volume were anlayzed. Scanning electron microscope (SEM) images were recorded after Pt coating by using cold type SEM, Hitachi S-4800, Japan. Transmission electron microscope (TEM) study of nanosheets was carried out by FEI Tecnai $G^2$ F30 S-Twin operated at 300kV. For TEM examination, a drop of nanosheets suspended in solvating agents was deposited on a TEM grid (ultrathin carbon film on lacey carbon support, 300 mesh Cu) and all samples were dried in a vacuum oven at 50 °C overnight.

## Notes and References


1. Choi, M.; Na, K.; Kim, J.; Sakamoto, Y.; Terasaki, O.; Ryoo, R. Stable single-unit-cell nanosheets of zeolite MFI as active and long-lived catalysts. *Nature* **2009**, *461*, 246.
2. Swei, J.; Talbot, J.B. Development of high-definition aqueous polyvinylpyrrolidone photoresists for cathode ray tubes. *J. Appl. Polym. Sci.* **2006**, *102*, 1637–1644, doi:10.1002/app.23950.
